# Biomonitoring with the Use of the Herbal Plant *Taraxacum officinale* as a Source of Information on Environmental Contamination

**DOI:** 10.3390/plants13131805

**Published:** 2024-06-29

**Authors:** Zuzanna Respondek, Oznur Isinkaralar, Paweł Świsłowski, Kaan Isinkaralar, Małgorzata Rajfur

**Affiliations:** 1 Institute of Environmental Engineering and Biotechnology, University of Opole, B. Kominka St. 6, 6a, 45-032 Opole, Poland; zuzares5@gmail.com; 2 Department of Landscape Architecture, Faculty of Engineering and Architecture, Kastamonu University, 37150 Kastamonu, Türkiye; obulan@kastamonu.edu.tr; 3 Institute of Biology, University of Opole, B. Kominka St. 6, 6a, 45-032 Opole, Poland; pawel.swislowski@uni.opole.pl; 4 Department of Environmental Engineering, Faculty of Engineering and Architecture, Kastamonu University, 37150 Kastamonu, Türkiye; kisinkaralar@kastamonu.edu.tr

**Keywords:** bioindicator, dandelion, metals, soil, pollution, bioconcentration factor, atomic absorption spectrometry

## Abstract

The aim of this study was to assess the level of contamination of the common dandelion—*Taraxacum officinale*—with selected metals (Mn, Fe, Ni, Cu, Zn, Cd, and Pb) and to demonstrate that this plant can be used in passive biomonitoring of industrial sites. Two sample transects (the first was near a forest, an area potentially uncontaminated by analytes [A], while the second ran near a steel mill, a contaminated area [B]), each about 1.5 km long, located in Ozimek, Opole Province, Poland, were used in this study. Metals in plant and soil samples were determined by atomic absorption spectroscopy (AAS). Based on the analysis of the obtained results to determine the concentration of metals, plants at site A were more contaminated with Mn (240 mg/kg d.m.) and those at site B with Fe (635 mg/kg d.m.). Mean Pb values (8.39 mg/kg d.m.) were higher at the industrial site (B) and statistically significant at the forest site (A), together with Mn and Fe at the *p* < 0.001 level. The *BCF* values for *T. officinale* showed that Cu (0.473) and Zn (0.785) accumulated to an average degree on both transects. This shows that dandelion is heavily loaded with these metals. Both dandelion and soil samples showed the highest concentrations of Mn, Fe, and Zn, especially in the polluted area B, which is the result of pollution not only from the smelter (dust from electric arc furnaces in steel smelting, extraction installations in production halls transmitting pollutants into the air from molding sand, or waste from molding and core masses dumped on the heap and blown by the wind from the landfill) but also from the high anthropopressure caused by human activity—for example, heating processes or road transport. Our results confirmed that *Taraxacum officinale* can be successfully used as a herbal plant in passive biomonitoring to assess the quality of the environment, but it must be collected from uncontaminated areas if we want to use it like a medicinal plant.

## 1. Introduction

A wide variety of plant and animal organisms are used for biomonitoring (bioindication) studies due to their response to changes in the state of the environment—through anatomical, morphological, or physiological processes that occur as a result of the pollution of a given ecosystem. Each bioindicator should exhibit widespread occurrence, easy availability, simple recognition, and high tolerance for specific factors. The level of metal contamination in urbanized areas is most often analyzed using mosses, lichens, mushrooms, numerous tree species, or herbaceous plants, which have the advantage of being cheap and simple to study [1,2,3,4]. The suitability of plants, and especially herbs, for use as bioindicators has been established and proven in many studies. The main species used in environmental biomonitoring include common dandelion (*Taraxacum officinale*), common nettle (*Urtica dioica*), and broadleaf plantain (*Plantago major*) [5]. The relationship between plants and soil in terms of the uptake of not only nutrients but also metals that exist as hazardous substances in the environment is becoming the basis for bioindication methods when assessing environmental pollution by these compounds [6].

The best metal uptake/accumulation potentials were noted in a relatively small stock of plants, known as hyperaccumulators. The vast majority of hyperaccumulator plants identified are mainly from a group of wild herbaceous species, usually restricted to their native habitats, with shallow root systems, slow growth rates, and low biomass yields. These plant species are typically small weeds that can grow on metal-bearing soils without showing negative toxic effects and tolerate and concentrate significant amounts of metals in their above-ground parts. Hyperaccumulator plants are also characterized by a high bioaccumulation factor (*BAF*). All hyperaccumulator species have *BAF* values much higher than 1, which essentially means that they can extract, translocate, and concentrate metals very efficiently and practically at levels much higher than the corresponding levels in the soil [7]. The ability of plants to develop resistance to metals in the soil is genetically determined and can be adaptively stimulated in specific situations in nature. Indicator species can be beneficial for planting and revitalizing areas around mines, industrial plants, or other areas where the soil is contaminated with metals [8]. In order to properly record and assess the metal load of a study area, it is necessary to use the appropriate statistical analysis tool. For the study of As and metal content in soils in Greece, a three-year study was carried out to investigate possible temporal changes in As and metal levels. The levels of As and metals determined were below the maximum permissible levels, except for Cd. No statistical differences were observed between the years of the study, although a trend of a continuous increase in their content was detected [9]. Understanding the spatial distribution of metals in soils is important for public health, spatial planning, source identification, and risk mitigation. The integration of GIS-based approaches and multivariate or spatial statistical analysis techniques is valuable in performing such spatial studies [10]. In large cities, green parks also have a significant impact on metal contamination, which, to varying degrees, can affect human health through air, water, or other sources. Therefore, it is becoming increasingly important to study the elemental content of soil and vegetation in urban parks and assess the risk of metal contamination. The ability of plants to uptake metals and how they are distributed in soil and plants allows for the observation of the behavior of contaminants. This provides the possibility of predicting their impact on park or green space ecosystems [11].

Biological monitoring, involving the common dandelion as a bioindicator of environmental quality, is comprehensively carried out worldwide. This shows activity to accumulate in tissues (mainly through leaves and roots), the large amount of pollutants present in the air and soils (near urban traffic routes, industrial plants, and rail transport) [1,3], so it can also participate in their phytoremediation in areas contaminated with metals [12]. A high relative accumulation rate of certain pollutants characterizes the dandelion. It meets all the criteria adopted for indicator plants: it is very common worldwide and easy to identify, and it is easily adapted to different geomorphic, soil, and climatic conditions [13]. An analysis of the literature shows that dandelion has been used in many studies as the preferred plant species for determining the level of environmental pollution based on the metal content of its tissues [14]. *Taraxacum officinale* is used actively in phytoindicative biogeochemical studies. The plant grows in a wide range of temperatures and meteorological conditions, as well as in sites with different levels of pollution; it is easily identified, and sampling is easy and economically viable. Dandelion tolerates a wide range of environmental conditions and is, therefore, a biomonitor of ecosystem contamination with metals such as Cd, Cr, Cu, Fe, Ni, Ti, Mn, Pb, and Zn [15]. It contains several pharmacologically active compounds—for example, flavonoids, phenolic acids, terpenoids, triterpenes, and sesquiterpenes. Its roots are a useful liver stimulant, while its leaves show diuretic and choleretic effects [16]. Dandelion is found in temperate regions and is commonly regarded as a ubiquitous weed in pastures, meadows, lawns, and roadsides, but in some areas, it is also used for phytopharmaceutical or nutritional purposes. In addition, it shows a high tolerance to toxic substances, drought, and nutrient deficiencies. These characteristics make it suitable for metal accumulation and biomonitoring studies [17].

High levels of metals in medicinal plants and in the soil that are due to pollution caused by anthropogenic sources (especially traffic-transmission of car exhaust fumes), population growth, urbanization, and industrialization are becoming an increasing global problem. Plants (especially medicinal plants and their products) growing in a polluted environment can accumulate metals in high concentrations, causing serious risks to human health. Therefore, it is necessary to monitor food quality, given that plant uptake is one of the main routes through which metals enter the food chain [3,18]. The use of plants as organisms capable of responding to any environmental contamination before it affects humans allows toxicological effects to be identified [19]. By monitoring these pollutants spatially, it is possible to interpret soil or atmospheric deposition sources in terms of land use in urban areas [20].

The aim of this study was to assess the level of contamination of common dandelion (*Taraxacum officinale*) with selected metals (Mn, Fe, Ni, Cu, Zn, Cd, and Pb) and to demonstrate that this plant can be used in passive biomonitoring of industrial sites (collected from different ecosystems), and the concentrations can be analyzed with a view to its use as a medicinal plant. The novelty of this article relates to the inclusion of herbal plants in biomonitoring studies in urban areas; these have not been extensively studied in the area to date. We determined elemental concentrations and identified sources of contamination, together with an assessment of their possible use for medicinal purposes.

## 2. Results and Discussion

### 2.1. Statistical Analysis

The tables below (Table 1 and Table 2) illustrate the concentration of individual elements for dandelion and soil samples at transects A and B.

On the basis of the tests carried out, it was shown that, in dandelion, the mean elemental concentrations (mg/kg d.m.) at the unpolluted site (A) follow the descending series Fe > Zn > Mn > Cu > Pb > Ni > Cd, and, on the contaminated site (B), Fe > Zn > Mn > Pb > Cu > Cd > Ni. In the dandelion, an almost twofold increase in metal concentrations (except Ni) is observed in the area near the smelter, compared with the forested area. The highest values of individual metals in *T. officinale* on transect A were found in samples located near the forest, close to a housing estate and single-family houses, which may be related to the burning of wood or other waste in the meadow area near the forest, close to the expanding housing estate. An increase in these metals may be caused by the progressive construction of houses and by other tasks carried out by workmen. In the forest area near a traffic road, the increase may be caused by car exhausts and the abrasion of car surfaces or tires. The lowest concentrations were determined in the area of the meadow near the forest. In the contaminated area (B), the highest concentrations of metals in dandelion were in the area of the allotment gardens, in the area near the smelter, in the green area near the still-active part of the smelter tailings pile, and in the area of the traffic road close to the former decommissioned smelter waste dump; the lowest were in the forest area behind the allotment gardens and outside the smelter, in the area of the allotment gardens, and close to the traffic road and the petrol station.

Comparing the results obtained with current scientific publications, it can be concluded that, for dandelion, the mean concentrations of Fe, Ni, Cd, and Pb are higher and, in the case of Cu and Zn, lower for both sites when compared with studies conducted in the town of Varaždin in northern Croatia [21]. The mean values of metal concentrations in *T. officinale* leaves, collected in rural and industrial areas in Italy [22], are also higher for Mn, Fe, and Cu and lower for Zn, Cd, and Pb in both zones (A and B), compared with the results in this paper. In the authors’ data from a study conducted in the war zones of eastern Croatia [23], it also appears that only Fe has lower average concentrations, with the other elements (Ni, Cu, Zn, Cd, and Pb) exceeding the level of these quantities. For other herb species, namely chamomile (*Matricaria chamomilla* L.), ribwort plantain (*Plantago lanceolata* L.), lemon balm (*Melissa officinalis* L.), peppermint (*Mentha* x *piperita* L.), and other plants described in articles [24,25], the results are lower for all herbs for Zn [24] and higher for Fe, Ni, and Cu [25]. The other metal concentrations (Mn, Cd, and Pb) presented in these publications [24,25] differ little from those presented in the article. In the next table (Table 2), we present the results for the concentration of elements in soil samples from two transects.

Soil samples showed the highest mean concentrations of Fe, Mn, and Zn and the lowest of Pb, Cu, and Ni in both the unpolluted (A) and polluted (B) sites. Higher concentrations of Ni, Cu, and Pb were also determined in the soil samples compared with the dandelions. Metal concentrations are almost six times higher in the soil samples than the dandelions taken from transect B. In soil, the highest concentrations of elements in the unpolluted area were in the samples located beside the forest near the carpentry—the production workshop, in the vicinity of the shooting range in the area of the forest (the increase in analyte concentrations may be due to the use of firearms), and the housing estate and single-family houses; the lowest were in the area of the meadow near the entrance to the forest. In the soil in the contaminated area (B), the greatest increase in Fe concentrations was found in the forest area outside the smelter, in the zone close to the smelter, and in the green area near the remaining section of the smelter heap; the smallest increase was in the green area behind the allotments and in the meadow and arable field sectors. The high concentrations of individual metals, especially Mn, Fe, and Zn, in the contaminated area (B) may be the result of emissions to the environment (atmospheric aerosol) of dust from the steelworks, which originate from electric arc furnaces used in steel smelting, from extraction systems in the production halls transmitting pollutants into the air, from molding sand, or waste from molding and core sand dumped on the heap and blown by the wind from the landfill, as well as from heating or traffic processes (car exhaust).

In the case of the results from the soil samples, the average concentrations of Mn, Cu, Zn, and Pb are much higher, except for Fe and Cd (slightly lower), compared with studies that have been conducted by other authors investigating the accumulation of these metals in soil and dandelion [16]. For the data obtained at both sites, we have the opposite situation to the article describing the average values of metals in soil samples collected in Varaždin in northern Croatia and in Italy [21,22], as the average concentrations of Fe and Cd are much higher, and Mn, Cu [21,22], Ni, Zn, and Pb are lower [21] compared with those presented in the cited article. In the soil samples, the contents of all metals are higher in both areas (A and B) than in the studies on chamomile and plantain, taken from different sites (meadow, brickyard, roundabout, park, mine, field) [24], and lower for Ni, Cu, and Pb from soil samples collected from the experimental plot located in Opole [25]. According to the conclusions of the authors of the study in northern Croatia, the main cause of air pollution by metals is the combustion of fossil fuels and road transport (exhaust fumes emitted into the atmospheric aerosol), while soils are polluted by surface or soil runoff following heavy precipitation [21]. It is also important for the analysis and interpretation to take into account the statistical significance of the data, as shown in the next table (Table 3).

Less than 43% of the cases showed statistically significant differences (see Table 3). Three elements showed a statistically significant difference in terms of concentrations in the plant between the two sites. For Ni and Cd, no determination could be made, and the concentrations of Cu and Zn were not statistically dependent on the sampling site. Exactly the same elements, namely Mn, Fe, and Pb, showed statistically significant differences in terms of their concentration in soil depending on the sampling site. For the other cases, this significance could not be determined (Cd, see Table 3) or the differences were not statistically significant at all. It should be noted that the statistically significant differences for individual elements were of a different nature in plants and soil (see Table 1 and Table 2). The literature reports variability in concentrations depending on the sampling site [26,27]. For *T. officinale*, it was found that the amount of metals measured in soils and plants corresponded to the contamination load of the sampling site. It should also be considered that the distribution of metals in dandelion depends on the total content and amount of available metal forms in the soil [13]. The concentration in the plant will also depend on the time of sampling [19,28]. Meteorological conditions, soil parameters, type of grassland, and landscape structure are also influential factors [29]. Plant morpho-physiological parameters also change due to traffic intensity and distance from the road—the average plant weight in a high-traffic area and the chlorophyll content in the same area showed a strong positive correlation [15].

Following the Regulation of the European Parliament and of the Council (EC) of 16 December 2008, the maximum permissible concentrations of the determined analytes in the soil were not exceeded [30]. In the above-ground parts (leaves, stems) of dandelion, the concentrations of Pb and Cd in individual samples exceeded the permitted values for herbs in the Commission Regulation (EU) of 25 April 2023 [31]. This means that the herbs taken from the study area should not be used for therapeutic/medicinal purposes. Table 4 shows the average *BCF* values and the degree of accumulation of specific elements.

Analyzing the *BCF* values shown in Table 4, Cd accumulated most intensively in transect B, while Ni, Cu, Zn, and Pb accumulated to an average degree in dandelion at both sites. This shows that dandelion takes up these metals to a moderate, medium degree from the soil. Fe was the least accumulated element in the herbs in area A, and Mn in area B. The *BCF* value in the unpolluted area was 43% higher than in the polluted area. The plant species studied showed the highest bioavailability and efficiency for bioaccumulation from the soil of Cd (B), Ni, Cu, Zn, and Pb (sites A and B), and the lowest for Fe (A) and Mn (B). Slightly higher results (except for Fe in Zakopane—0.023 and in Siedlce—0.021) were obtained in a 2018 study by Królak et al. on metal accumulation in dandelions, sampled from southern (Zakopane) and eastern (Siedlce) Poland (for Mn: Zakopane—0.26, Siedlce—0.16; for Cu: Zakopane—1.67, Siedlce—2.24; for Zn: Zakopane—0.54, Siedlce—0.78) [32].

Natural Pb concentrations in dandelion leaves are approximately 0.2 μg/g. This value is 10–1000 times lower than most reported measurements of modern Pb concentrations in dandelion leaves [33]. Pb is an element that can enter plants via soil or air. The normal content of this metal in plants ranges from 0.1 to 10 mg/kg. The toxic Pb content ranges from 30 to 300 mg/kg [34]. Therefore, the Pb concentrations we obtained against natural concentrations indicate that the exposure of samples to negative pollutants is proportional to urbanization, industrial activity, and traffic density [14]. In many cases, the average levels of the metals tested (such as Fe) from sites with more intense anthropogenic pollution were statistically significantly higher than in other areas [35].

### 2.2. Spatial Distribution

**Figure 1:**
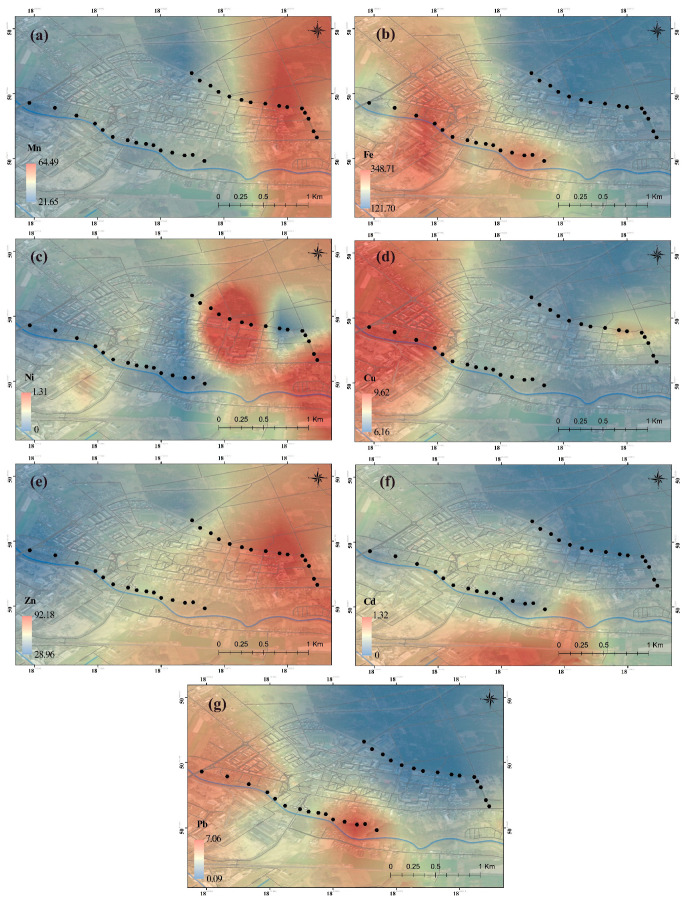
Spatial distribution of (**a**) Mn, (**b**) Fe, (**c**) Ni, (**d**) Cu, (**e**) Zn, (**f**) Cd, and (**g**) Pb in the plant. Black dots represent the sampling sites.

**Figure 2:**
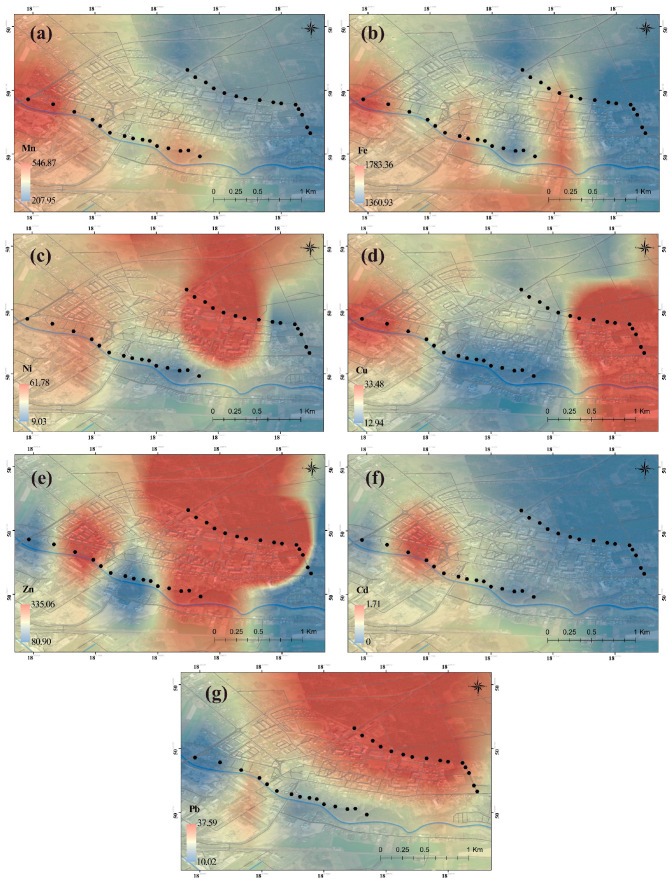
Spatial distribution of (**a**) Mn, (**b**) Fe, (**c**) Ni, (**d**) Cu, (**e**) Zn, (**f**) Cd, and (**g**) Pb in the soil. Black dots represent the sampling sites.

The maps obtained as a result of the visualizations can be found in Figure 1 (in the plant) and Figure 2 (in the soil).

Although Fe, Ni, Zn, and Cd concentration values may be higher or lower, it was observed that they exhibit similar behavior spatially. Due to atmospheric deposition, Mn accumulation is high in the soil in the west and in the plants in the east. The Cu concentration is intense in the plant in the west of the research area. On the other hand, high values were found in the soil in the east. Pb accumulation is observed differently in plants and soil in the south and southwest. This finding suggests that there may be an east-oriented source for Mn, a west-oriented source for Cu, and a south-southwest-oriented source for Pb in plants. Wind is one of the factors influencing the distribution of dust particles in the atmospheric aerosol and the concentration of metals in samples [36,37]. The wind rose for the area, which indicates that winds mainly blow from the W and SW directions [38]. However, it should be remembered that the concentration of metals in samples is also influenced by the local pollution sources [39], which is also connected to medicinal plants being under threat from anthropogenic activities and land use/cover changes [40].

## 3. Materials and Methods

### 3.1. Study Site

**Figure 3:**
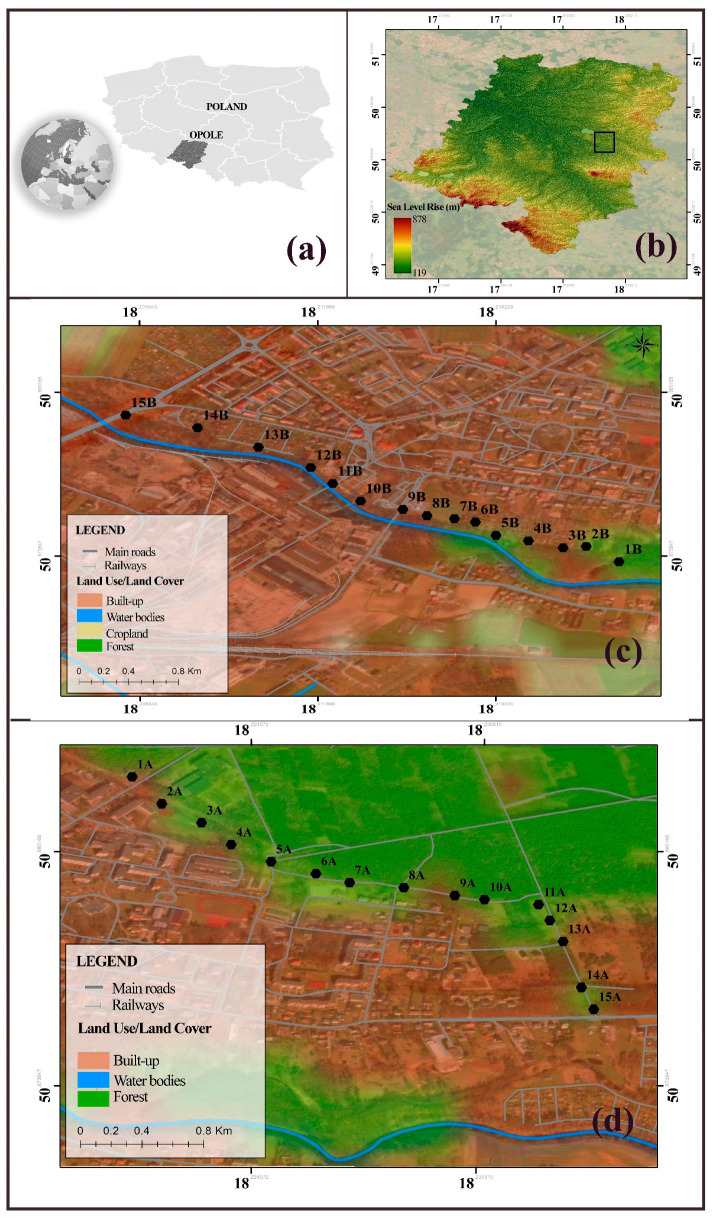
Location of the study area: (**a**) in relation to Poland; (**b**) measurement site in the Opolskie Voivodeship; (**c**) distribution of the individual survey points [black dots] in transect B [contaminated area]; (**d**) transect A [uncontaminated area].

The study, using *Taraxacum officinale*, included two sample transects of about 1.5 km in the Opole Province in Poland (in the town of Ozimek, 21 km from the city of Opole). The location of the transects and the distribution of the individual survey points are shown on the maps in Figure 3. Exact GPS locations are presented in the Supplementary Materials in Table S1. The town of Ozimek is surrounded by deciduous and coniferous forests, which are part of the Stobrawskie Forests, and is located at a distance of 10 km from the complex of the three Turawskie lakes. The Mała Panew River, which is the right tributary of the Oder River, flows through the southwestern sector of the town. The town has an industrial plant—ironworks—which was established in 1754 [41,42]. Currently, there are no major mineral deposits in the Opolskie Province, with the exception of rock, which includes limestone, marl, granite, and basalt. These types of rock are exploited in larger quantities in the central part of the province, Kotlarnia, and the Opawskie Mountains. In the area of the Opolskie Province, there are twelve groups of raw materials coming from 268 documented rock deposits with a total balance of resources of 3.977 billion Mg, which constitute 7.1% of the national resources. Approximately 29% of these resources are industrial resources, with a national share of 11%. The largest share of the national industrial resources is in the form of backfill sands—45%, limestone for the cement industry—28.1%, and limestone for the lime industry—24.5%. Deposits of various types of iron ore are common in Poland but are usually characterized by their small size and contain iron-poor ores [43]. Therefore, they have not been mined in Poland since the last decade of the 20th century, although they were mined for domestic metallurgy, construction, and other industries in the past [44]. Due to the industrial nature of the study site, this area has already been covered by biomonitoring studies [45], so our research is a continuation of the research in the area.

The first transect was located close to the forest (designated in the article as a potentially uncontaminated area with metals—transect A), while the second ran close to the smelter (contaminated area—transect B). In both transects, 15 study sites were mapped (30 survey points in total). The test sites were approximately 100 m apart. The wind direction most frequently observed in the study area was from the west and southwest [38].

### 3.2. Passive Biomonitoring and Analytical Procedures

Passive biomonitoring was applied by collecting 30 dandelion samples (i.e., the green parts—leaves and stems) and 30 soil samples (surface soil layer up to 10 cm). Sixty samples were taken in total from designated sampling points lying in two transects differing in levels of metal contamination. Samples of material were collected in early May 2022. The collected material was dried at a temperature of approximately 100 °C for 24 h to obtain dry mass (d.m.). The samples thus prepared were crushed in a mortar and stored in sealed polyethylene containers. Soil samples were sieved after being crushed to a specific mesh size of 2 mm. Subsequently, representative (averaged) dandelion samples weighing 0.400 ± 0.001 g were mineralized. The samples were flooded with a mixture of concentrated nitric acid (V) HNO_3_ 65% and perhydrol H_2_O_2_ 30% in a 3:1 ratio and left for 15–20 min for pre-digestion [45]. After this time, the samples were placed in a Speedwave Four microwave mineralizer from BERGHOF, Germany. The mineralization process was carried out at 180 °C for 45–50 min. A representative (averaged) soil sample weighing 0.500 ± 0.001 g was mineralized. The samples were flooded with a mixture of concentrated nitric acid (V) HNO_3_ 65% and hydrochloric acid HCl 35% (aqua regia) in 10 mL and left for 15–20 min for pre-digestion. After this time, the samples were placed in a Speedwave Four microwave mineralizer from BERGHOF, Germany. The mineralization process was carried out at 180 °C for 60–65 min. After mineralization, the solutions were filtered and diluted into 20 cm^3^ (herbs) and 25 cm^3^ (soil) volumetric flasks with demineralized water (conductivity κ = 0.5 µS/cm). The solutions were prepared using Merck reagents. The next step was the determination of Mn, Fe, Ni, Cu, Zn, Cd, and Pb concentrations in the mineralized samples by atomic absorption spectrometry (AAS), using an iCE 3500 atomic absorption spectrometer from ThermoElectron Corporation, West Palm Beach, FL, USA.

Table 5 shows the limits of quantification of metals characterizing the iCE 3500 spectrometer [46]. Calibration of the instrument was carried out using standards from ANALYTIKA Ltd., Prague, Czech Republic. The values of the highest concentrations of standards used for calibration (2.0 mg/dm^3^ for Cd; 5.0 mg/dm^3^ for Ni, Cu, Zn, and Pb; 7.5 mg/dm^3^ for Mn; and 10 mg/dm^3^ for Fe) were taken as the limit of the linear dependence of the signal on concentration.

### 3.3. Quality Control

Table 6 shows the concentrations of elements determined in certified reference materials BCR-414 *plankton* and BCR-482 *lichen*, produced by the Institute for Reference Materials and Measurements, Belgium, as a source of quality control for our measurements.

### 3.4. Data Processing and Statistics

In order to assess the bioaccumulation properties for the individual analytes in the herb samples, the bioconcentration factor (*BCF*) (i.e., the ratio of metal concentration in the plant to the concentration in the soil) was determined according to the following formula [48]:

$$BCF=\frac{C_{p}}{C_{s}}$$

where *c_p_*—metal concentration in the overground part of the plant [mg/kg d.m.], and *c_s_*—available metal concentration in the soil [mg/kg d.m.].

*BCF* values have been interpreted as follows: *BCF* < 0.01—no accumulation; *BCF* < 0.1—weak degree of accumulation; *BCF* < 1.0—medium degree of accumulation; *BCF* > 1.0—intensive degree of accumulation.

Spatial distribution maps were produced using ArcGIS 10.4.1 software to spatially understand the accumulation of metals in plants and soil and relate them to land use [49]. 

The statistics were performed using Microsoft Excel 2021 and STATISTICA (version 13.3) software to process and present the data. For descriptive analysis, the minimal and maximal values, median, and means were calculated for each analyzed element (see Figure 2). The significance of the differences between locations was provided by independent pooled samples [50]. The normality of the data was tested by the Kolmogorov–Smirnov test due to sample sizes [51]. Differences between the locations in terms of elemental concentrations in the herbs and soil samples were evaluated by the parametric Student’s t-test and nonparametric Mann–Whitney U test, respectively. In order to avoid substantial differences between the concentrations of the determined elements, which leads to the nonnormal distribution of the data, the Box-Cox transformation was used to improve and boost the normality of the data [52]. The difference was considered statistically significant at *p* < 0.05.

## 4. Executive Summary and Conclusions

The analysis of metal content in the environment by means of biomonitoring, using medicinal plants, can give information on the level of pollution and allow the sources of these analytes to be determined, especially in urbanized areas. The use of the common dandelion (*Taraxacum officinale*) as a bioindicator of the state of the environment made it possible to determine the amount of pollutants in the atmospheric aerosol in the study area—a forested area (unpolluted—A) and a steelworks area (polluted—B), located in Ozimek (Opole Province). To continue this research in the future, some geochemical indices can show the genetically inherited trace element input in soil samples and how much of the pollutants are of anthropogenic origin.

The following conclusions were drawn from the study:

(1) The concentrations of metals were almost six times higher in soil samples than in dandelion, which is due to the slow process of phytoremediation of soils from metals as well as pollution caused by human anthropogenic activities. The highest concentrations in soil were noted for Fe at 1482 mg/kg d.m. and 1670 mg/kg d.m. in areas A and B, respectively. Nevertheless, in accordance with the Regulation of the European Parliament and of the Council (EC) of 2008, the maximum permissible concentrations of the determined analytes in soil were not exceeded.
(2) In the above-ground parts (leaves, stems) of dandelion, the concentration of Pb and Cd in individual samples exceeded the permitted values given for herbs in the 2023 Commission Regulation.
(3) The dandelion itself and the soil showed the highest concentrations of Mn (52.4 mg/kg d.m.), Fe (635 mg/kg d.m.), and Zn (143 mg/kg d.m.), especially in the contaminated area (B), which is the result of pollutant emissions from the smelter (dust from electric arc furnaces in steel melting, extraction installations in production halls transmitting pollutants into the air from molding sand, or waste from molding and core sand dumped on the heap and blown by the wind from the landfill), but also of high anthropopressure caused by human activity—for example, heating processes or road transport (car exhaust fumes).
(4) The *BCF* values for *Taraxacum officinale* showed that Cu and Zn accumulated to a medium degree at both sites A and B, with values from 0.452 to 0.785 [-]. The implication is that dandelion takes up these metals to a moderate to medium degree from the soil.
(5) The study confirmed that the analyzed plant can be used as a bioindicator in passive biomonitoring to assess the degree of environmental pollution by selected elements (Mn, Fe, Ni, Cu, Zn, Cd, and Pb).
(6) Medicinal/herbal plants should only be taken from potentially clean (unpolluted) areas, unencumbered by human anthropogenic activity, for therapeutic/food purposes.

## Figures and Tables

**Table 1:** Concentration of metals in *Taraxacum officinale* in two studied areas [mg/kg d.m.].

Plant—Site A			
Element	Min–Max	Mean	Median
Mn	9.46–240	49.1	28.4
Fe	82.0–207	133	138
Ni	2.63–4.94	3.79	3.79
Cu	2.44–9.88	6.52	7.05
Zn	25.7–161	68.1	58.2
Cd	1.04–1.66	1.35	1.35
Pb	3.61–7.00	5.14	4.81
**Plant—Site B**			
**Element**	**Min–Max**	**Mean**	**Median**
Mn	15.9–52.4	31.1	31.9
Fe	102–635	281	257
Ni	2.55–2.55	2.55	2.55
Cu	3.80–9.78	7.50	7.98
Zn	14.2–143	57.0	49.7
Cd	0.830–5.91	2.48	1.96
Pb	3.70–20.3	8.39	6.96

**Table 2:** Concentration of metals in soil in two studied areas [mg/kg d.m.].

Soil—Site A			
Element	Min–Max	Mean	Median
Mn	86.6–605	264	220
Fe	1150–1804	1482	1408
Ni	3.56–138	22.1	8.61
Cu	6.96–64.3	19.9	14.2
Zn	34.9–388	147	107
Cd	n.d.	n.d.	n.d.
Pb	10.4–48.8	30.9	34.0
**Soil—Site B**			
**Element**	**Min–Max**	**Mean**	**Median**
Mn	204–752	382	347
Fe	1366–1998	1670	1780
Ni	7.23–37.1	16.7	10.4
Cu	8.70–27.0	17.0	15.7
Zn	61.5–353	147	119
Cd	0.890–3.42	1.91	1.61
Pb	5.89–54.1	20.3	20.4

n.d.: no data.

**Table 3:** Statistical significance of elemental concentrations in two studied areas.

Plant		
Element	Test	*p*
Mn	B-C	***
Fe	**S-T**	***
Cu	M-T	n.s.
Zn	S-T	n.s.
Pb	B-C	***
**Soil**		
**Element**	**Test**	***p***
Mn	**M-T**	**
Fe	**S-T**	*
Ni	M-T	n.s.
Cu	M-T	n.s.
Zn	M-T	n.s.
Pb	**M-T**	*

S-T: Student *t*-test; M-T: Mann–Whitney U test; values in bold are statistically significant at the level: * *p* < 0.05, ** *p* < 0.01, *** *p* < 0.001; n.s.: no statistically significant differences; B-C: Box-Cox transformation.

**Table 4:** *BCF* values for two areas.

Element—Area A	*BCF* Value [-]	Description
Mn	0.221	medium
Fe	0.092	weak
Ni	0.405	medium
Cu	0.473	
Zn	0.785	
Pb	0.249	
**Element—Area B**	***BCF*** **Value [-]**	**Description**
Mn	0.088	weak
Fe	0.171	medium
Ni	0.148	
Cu	0.483	
Zn	0.452	
Cd	3.220	intensive
Pb	0.637	medium

**Table 5:** Limits of detection (*IDL*) and quantification (*IQL*) for the iCE 3500 atomic absorption spectrometer from ThermoElectron Corporation (USA) [mg/dm^3^] [46].

Metal	*IDL*	*IQL*
Mn	0.0016	0.020
Fe	0.0043	0.050
Ni	0.0043	0.050
Cu	0.0045	0.033
Zn	0.0033	0.010
Cd	0.0028	0.013
Pb	0.0130	0.070

**Table 6:** Comparison of measured and certified concentrations in BCR-414 *plankton* and BCR-482 *lichen* [47].

	BCR-482 *lichen*		AAS		*Rel.* **
Metal	Concentration	±Uncertainty	Mean	±*SD* *	
	[mg/kg d.m.]				[%]
Mn	33.0	0.5	31.7	0.68	−3.9
Fe	804	160	771	154	−4.1
Ni	2.47	0.07	2.16	0.32	−13
Cu	7.03	0.19	6.63	0.17	−5.7
Zn	100.6	2.2	95.1	2.3	−5.5
Cd	0.56	0.02	0.53	0.03	−5.3
Pb	40.9	1.4	38.2	1.0	−6.6
	**BCR-414 *plankton***		**AAS**		*Rel.* **
**Metal**	**Concentration**	**±Uncertainty**	**Mean**	±*SD* *	
	[mg/kg d.m.]				[%]
Mn	299	12	284	13	−5.0
Fe	1.85	0.19	1.79	0.20	−3.2
Ni	18.8	0.8	18.2	0.9	−3.2
Cu	29.5	1.3	28.4	1.6	−3.7
Zn	112	3.0	107	3	−4.5
Cd	0.383	0.014	0.371	0.018	−3.1
Pb	3.97	0.19	3.75	0.21	−5.5

* standard deviation; ** relative difference between the measured (cm) and certified (cc) concentrations: 100% * (cm-cc)/cc.

## Data Availability

The original contributions presented in the study are included in the article; further inquiries can be directed to the corresponding author.

## Supplementary Materials

The following supporting information can be downloaded at https://www.mdpi.com/article/10.3390/plants13131805/s1, Table S1: Location of measurement sites.
