# Supplementary material for: Biomonitoring with the Use of the Herbal Plant Taraxacum officinale as a Source of Information on Environmental Contamination
Source: Plants (Basel). 2024 Jun 29;13(13):1805. doi: 10.3390/plants13131805 (PMC11244324; doi:10.3390/plants13131805)
Supplement: Supplementary file 1 [file plants-13-01805-s001.zip › plants-3047204-supplementary.pdf]

**Table S1.** Location of measurement sites.

| Sampling site- Area A | GPS location |
|---|---|
| 1A | 50°40'56.1''N 18°13'16.8''E |
| 2A | 50°40'53.4''N 18°13'19.7''E |
| 3A | 50°40'51.5''N 18°13'23.6''E |
| 4A | 50°40'49.3''N 18°13'26.5''E |
| 5A | 50°40'47.6''N 18°13'30.4''E |
| 6A | 50°40'46.4''N 18°13'34.8''E |
| 7A | 50°40'45.5''N 18°13'38.1''E |
| 8A | 50°40'45.0''N 18°13'43.4''E |
| 9A | 50°40'44.2''N 18°13'48.4''E |
| 10A | 50°40'43.8''N 18°13'51.3''E |
| 11A | 50°40'43.3''N 18°13'56.6''E |
| 12A | 50°40'41.7''N 18°13'57.7''E |
| 13A | 50°40'39.6''N 18°13'59.0''E |
| 14A | 50°40'35.0''N 18°14'00.8''E |
| 15A | 50°40'32.8''N 18°14'02.0''E |
| **Sampling site- Area B** | **GPS location** |
| 1B | 50°40'24.3''N 18°13'21.4''E |
| 2B | 50°40'26.5''N 18°13'17.2''E |
| 3B | 50°40'26.3''N 18°13'14.2''E |
| 4B | 50°40'27.3''N 18°13'09.8''E |
| 5B | 50°40'28.1'N 18°13'05.6''E |
| 6B | 50°40'30.0''N 18°13'03.0''E |
| 7B | 50°40'30.5''N 18°13'00.1''E |
| 8B | 50°40'30.9''N 18°12'56.8''E |
| 9B | 50°40'31.8''N 18°12'53.7''E |
| 10B | 50°40'33.0''N 18°12'48.3''E |
| 11B | 50°40'35.5''N 18°12'44.7''E |
| 12B | 50°40'37.8''N 18°12'41.9''E |
| 13B | 50°40'40.7''N 18°12'35.2''E |
| 14B | 50°40'43.5''N 18°12'27.4''E |
| 15B | 50°40'45.3''N 18°12'18.2''E |
